# Genome wide transcriptional analysis of resting and IL2 activated human natural killer cells: gene expression signatures indicative of novel molecular signaling pathways

**DOI:** 10.1186/1471-2164-8-230

**Published:** 2007-07-10

**Authors:** Karen Dybkaer, Javeed Iqbal, Guimei Zhou, Huimin Geng, Li Xiao, Alexander Schmitz, Francesco d'Amore, Wing C Chan

**Affiliations:** 1 Department of Pathology and Microbiology, University of Nebraska Medical Center, Omaha, NE, USA; 2 Eppley Institute for Research in Cancer and Allied Diseases, University of Nebraska Medical Center, Omaha, NE, USA; 3 Department of Hematology, Aarhus University Hospital, Aarhus, Denmark; 4 Molecular Biology, Aarhus University, Aarhus, Denmark

## Abstract

**Background:**

Human natural killer (NK) cells are the key contributors of innate immune response and the effector functions of these cells are enhanced by cytokines such as interleukine 2 (IL2). We utilized genome-wide transcriptional profiling to identify gene expression signatures and pathways in resting and IL2 activated NK cell isolated from peripheral blood of healthy donors.

**Results:**

Gene expression profiling of resting NK cells showed high expression of a number of cytotoxic factors, cytokines, chemokines and inhibitory and activating surface NK receptors. Resting NK cells expressed many genes associated with cellular quiescence and also appeared to have an active TGFβ (TGFB1) signaling pathway. IL2 stimulation induced rapid downregulation of quiescence associated genes and upregulation of genes associated with cell cycle progression and proliferation. Numerous genes that may enhance immune function and responsiveness including activating receptors (*DNAM1, KLRC1 *and *KLRC3*), death receptor ligand (*TNFSF6 (FASL*) and *TRAIL*), chemokine receptors (*CX3CR1, CCR5 *and *CCR7*), interleukin receptors (*IL2RG, IL18RAB *and *IL27RA*) and members of secretory pathways (*DEGS1, FKBP11, SSR3, SEC61G *and *SLC3A2*) were upregulated. The expression profile suggested PI3K/AKT activation and NF-κB activation through multiple pathways (TLR/IL1R, TNF receptor induced and TCR-like possibly involving BCL10). Activation of NFAT signaling was supported by increased expression of many pathway members and downstream target genes. The transcription factor *GATA3 *was expressed in resting cells while *T-BET *was upregulated on activation concurrent with the change in cytokine expression profile. The importance of NK cells in innate immune response was also reflected by late increased expression of inflammatory chemotactic factors and receptors and molecules involved in adhesion and lymphocyte trafficking or migration.

**Conclusion:**

This analysis allowed us to identify genes implicated in cellular quiescence and the cytokines and cytotoxic factors ready for immediate immune response. It also allowed us to observe the sequential immunostimulatory effects of IL2 on NK cells improving our understanding of the biology and molecular mediators behind NK cell activation.

## Background

Natural killer (NK) cells are the crucial mediators of immune reponse against tumor cells and pathogens via modulating both innate and adaptive immune response [1-3]. They can either directly kill the tumor derived or virus infected cells, enhance the phagocytic or bactericidal activity of phagocytes or direct adaptive T cell response towards the Th1 pattern [4,5]. NK cells constitute around 10% of peripheral blood lymphocytes and are characterized phenotypically by surface expression of CD56 and CD16 but not CD3. The majority of NK cells (90%) are CD56^dim^CD16^bright ^– and mediate cytolytic activity against tumor or pathogen-infected cells whereas the remaining (10%) are cytokine producing NK cells with a CD56^bright^CD16^dim/negative^phenotype[6]. These NK subsets also express distinct chemokines that are important in their preferential localization within the lymphatic system [7].

The killing of target cells by NK cells is based on two alternative pathways, namely the perforin/granzyme secretory pathway and the death receptor pathway [8]. The secretory pathway can lyse target cells via spontaneous or antibody-dependent cell-mediated cytotoxicity (ADCC). Spontaneous cytotoxicity is initiated by ligand binding to activating NK receptors resulting in sequential recruitment and activation of SRC and SYK kinases, PI3K, RAC1, PAK1, MEK and ERK, leading to Perforin 1 and Granzyme B granule polarization and movement toward the ligated target cell [9]. ADCC killing involves the low affinity receptor for IgG on NK cells, CD16 (FcγRIII). The interaction of CD16 with IgG coating the target cells leads to signaling through FcεRIγ and CD3ζ resulting in cytotoxic granule polarization and possible activation of VAV, PI3K and PLC-γ2 [10]. The death receptor pathway is based on the interaction between NK surface-bound ligands such as TNFSF6 (FASL) and TRAIL or NK-secreted factors such as TNFα, LTA and LTB [11] with death receptors on the target cells thereby triggering a signaling cascade resulting in apoptosis of the targeted cells.

The cytokine producing NK cells are important in early innate immune response, where the release of cytokines and chemokines stimulates and recruits other cells. Response of the NK cells themselves is under the control of signaling events primed by activating and inhibiting surface receptors. These include different members of the human killer cell immunoglobulin-like receptors (KIR), the C-type lectin receptor families (Ly49 (KLRA1), CD159A (KLRC1), CD159c (KLRC2), NKG2E (KLRC3), CD94 (KLRD1), CLEC5C (KLRF1) and NKG2D (KLRK1)) and the natural cytotoxicity receptors (NKp46, NKp44 and NKp30) [12]. Immune response of NK cells is also under the influence of cytokines such as IFNα, IFNβ, IL2, IL12, IL15 and IL18 secreted by other cells of the immune system. IL2 is a pluripotent cytokine that can expand and activate NK cells [13] as well as promote their migration within target tissues [14], their cytotoxicity [15] and increase the secretion of cytokines, chemokines and other small molecules [16]. The molecular mechanisms underlying this classical NK cell activator for increased NK cell activity have not been elucidated.

We performed gene expression profiling on resting and *in vitro *IL2 activated human NK cells to increase our understanding of the molecular events important in NK cell activation. Increased knowledge of the mechanisms controlling chemokine and cytokine expression, cellular migration and IL2 mediated signal transduction pathways may help to enhance, re-direct or modify the immune and anti-tumor activity of NK cells and provide a valuable platform for the study of NK cell derived malignancies. We discuss the induction of novel signal transduction pathways during the NK cell activation and validate some of the differentially expressed genes by RT-PCR. The impact of the observed transcriptional profile in orchestrating the innate immune responeby NK cell is also discussed.

## Results

### General approach on time-dependent gene expression analysis of NK cells stimulated with IL2

**Figure 1:**
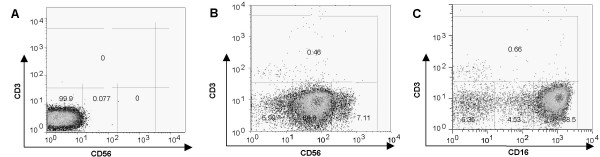
Flo**w **cytometry analysis of NK cells isolated from PBMC by negative selection. The purity of NK cells was assessed using fluorescence-labeled antibodies to CD3, CD16 and CD56. A. NK cells not stained with specific antibodies (isotype control). B. NK cells labeled with mAb for CD3 (Alexa 488) and CD56 (Alexa 647). C. NK cells labeled with mAb for CD3 (Alexa 488) and CD16 (Alexa 647). Less than 1% CD3 positive cells are present and > 94% of the cells are CD3- CD56+ while > 93% are CD3- and CD16 +.

The aim of the study was to investigate the activation of human NK cells by IL2 through analyzing the global gene expression at different time points (0, 2, 8 and 24 hours) after culture with the cytokine IL2 at 100 IU/ml. NK cells with the CD56^+^/CD16^+ ^and CD3^- ^phenotype were negatively selected by immunomagnetic beads and re-examined by flow-cytometry to ensure greater than 90% purity (Figure 1). Equivalent amount of total RNA from NK cells derived from 3 or 4 different donors for each time point was pooled, amplified and labeled before duplicate hybridizations was performed on spotted (pre-synthesized oligonucleotide) microarrays (additional file 1). To validate the gene expression data, a different set of experiments with 6 other individual donors was performed. RNA was similarly extracted and pooled from 4 different donors, amplified and labled according to the manufacturer's instruction (GeneChipU133plus2^®^, Affymetrix Inc, CA). The expression data generated from microarray experiments were uploaded in BRB-ArrayTool[17,18] to obtain the functional annotations of the geneID or probe sets and their normalized data were used for further analysis. Of the 54,676 probe sets represented on GeneChipU133plus2^® ^with 20,585 unique genes, 62 % (~12,750) of the genes were also represented on the spotted microarray. Only pathways showing a similar pattern of expression on the two platforms were selected for further analysis and a few of the genes were also validated by RT-PCR (Figure 5).

### Resting NK cell signature

The resting NK cell signature from the spotted microarray was defined as differentially expressed genes, that had ≥ 2 fold higher expressions in resting NK cells compared to the lymphoid RNA standard and also ≥ 2 fold higher expression than tonsillar cells and resting CD8^+ ^T cells. Thus, 1027 transcripts were included in the resting NK cell signature on the spotted array platform (additional file 2). The NK cell signature derived from the GeneChipU133plus 2^® ^was based on similar comparisons, with the modification that, tonsil profile was replaced with an universal RNA standard (Stratagene Inc, CA). In this format, 1133 unique transcripts were overexpressed in resting NK cells (additional file 3). Comparison of the NK cell signatures from the two platforms resulted in a common set of 164 genes (Table 1). The genes included inhibitory NK cell surface molecules, activating NK cell surface molecules as well as cytotoxic mediators. Many genes encoding chemokines, cytokines and their receptors and genes involved in secretory functions were also expressed at high levels in resting cells. We also examined a curated resting NK signature from the literature (S*ignature Data-base*) [19] derived from the Affymetrix platform [20]. Thirty-three of sixty genes (55%) were found in our Affymetrix derived signature, (additional file 4), demonstrating consistency within the individual platform.

**Table 1:** Resting NK signature derived from Affymetrix and spotted microarray platform.

		**Median fold change (Range)**	
**Gene ontogeny**	**Gene symbols**	**Spotted**	**Affymetrix**
**Cell cycle regulators**	*BTG1, JAG1, MATK, TGFBR3, TINF2, TMEPAI, NEK1, CCND2, CCNC, BIN1, HEXIM1, PPP2R5A, CARF*	**2.59 (2.01–5.06)**	**3.76 (2.87–7.05)**
**Cytotoxicity**	*CRTAM, CTSW, DPP8, FASLG, FNBP1, GNLY, GZMA, GZMH, HCST, LITAF, PRF1, TNFRSF1B, TNFSF14, NEK1, CASP10, STK17B, CTSC, PRSS23*	**4.48 (2.36–12.76)**	**4.43 (2.8–19. 42)**
**Cytokines**	*CCL5, CX3CR1, IL18R1, IL18RAP, IL21R, PPBP(CXCL7)*	**7.12 (2.9–17.56)**	**5.93 (2.86–29.7)**
**Inflammatory response**	*C3AR1, PDE7A, LY75, RORA, AOAH, ARF6, C1orf38, CLEC2B, IRF1*	**3.12 (2.20–6.56)**	**5.06 (2.83–11.01)**
**Regulators of immune response **	*PTPN4(negative regulators), PPP2R2B, SLA2, ID2, KLF11, ZFHX1B*	**3.18 (2.36–8.6)**	**9.92 (2.84–11.39)**
**Secretory pathway and protein maturation **	*GALNT11, GNPTAB, RAMP1, SEC22L3, SLC38A1, TES, TPST2, WDFY1, GALNT7, DNAJC3, SLC35E1, ZNF12*	**3.88 (2.90–9.01)**	**4.34 (3.00–4.96)**
**Transcription factors and accessory regulators **	*BHLHB2, EOMES, KBTBD2, MAF, NFAT5, THRAP3, TXK, ZBTB11, ZNF267, ZNF394, ZNF12, HCF2, MLLT10, MLL3, SSRPN, TAF1, MLR1, ZNF614, EIF2C4, EIF1AY, EIF2C2*,	**3.55 (2.58–7.44)**	**3.71 (2.88–4.9)**
**Regulation of GTPase activity**	*CENTD1, PLEK, PREX1, RAB7L1, RASGRP1, RGS18, RGS3, RIN3, PCTK2, RAB27B*	**4.16 (2.91–5.67)**	**4.43 (3.64–6.8)**
**Proliferative**	*GFI1, SMAD5, ZBTB16, AKT3, SMC5L1, RNF139*	**2.90 (2.90–22.16)**	**3.02 (3.02–5.3)**
**Cytoskeleton orgnisation**	*DOCK5, DOCK8, DOK2, MACF1, MYOM2, PHLDB2*	**3.41 (2.73–6.21)**	**3.51 (2.94–8.50)**
**Metabolism and nucleotide biosynthesis **	*DCY7, ATP6AP2, CHST12, CLIC3, GFOD1, PARP8, TKTL1, YPEL1, ENPP4, DHRS7, BPGM*	**2.92 (2.25–19.24)**	**4.98 (2.84–9.55)**
**Miscellaneous**	*SBK1, PLAC8, PLCL2, RNF125, SPAG9, MAPK1, C10orf137, C10orf18, C1orf63, C20orf3, FLJ13611, FLJ39739, GALNT10, ZBTB11, BTBD9, C14orf43, BRWD1, FAM62B, BCAP29, C18orf8, LOC285628, KLHL21, KIAA0907, KIAA1450, MOBKL2A, TSPYL1, TRIM39, ULK2, ULK4, WAC, YTHDC2, ZSWIM6*	**4.16 (2.42–10.13)**	**4.23 (2.94–8.17)**
**Cell adhesion and cell surface**	*ITGAM, ITGB2, ADAM8, FGR, CD300A(Irp60), CD3Z, CD96, FCER1G, CD94 (KLRD1), CD314 (KLRK1), LAIR2, ADD3, CD244, SPN(CD43), KLRF1*	**3.36 (2.36–8.9)**	**3.90 (2.4–21.53)**

Resting NK signature of genes showing 2 fold higher expression levels on two microarray platforms. A common set of 164 genes were more than 2 fold higher expressed in resting NK cells compared to 1) the lymphoid RNA standard, tonsillar cells and resting CD8^+ ^T cells on spotted microarrays and 2) the lymphoid RNA standard, universal RNA standard (Stratagene Inc, CA) and resting CD8^+ ^T cells on GeneChipU133plus 2^® ^(Affymetrix Inc, CA).

### Temporal analysis of the IL2 stimulated NK cells

Self organizing maps (SOM) generated from spotted microarray data identified the differentially expressed genes that had at least a 2-fold alteration in expression level between the time points analyzed (additional file 5). The expression of the selected pathways was examined and cross-validated on both platforms. Additional genes on Affymetrix microarray platform (GeneChipU133plus2^®^) belonging to functional groups that were determined to be differentially expressed were included for more comprehensive interpretation.

#### Cytolytic pathway

**Figure 2:**
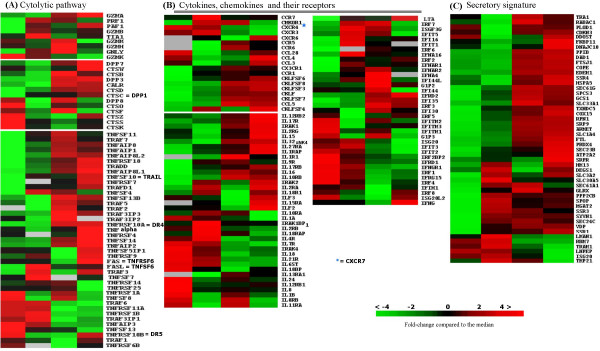
Functional groups of genes with maximal differences in expression levels between resting NK cells (column1) and NK cells after 2, 8 or 24 hours (colums 2,3 & 4 respectively) of IL-2 activation are shown. The color change in each row represents the gene expression relative to the median across the samples and values are visualized according to the scale bar that represents the expression fold difference (log2) relative to the median. A) Cytolytic pathway, B) Cytokines, chemokines and their receptors, and C) Secretory signature.

The mRNA of a number of molecules associated with cytolytic function were expressed in resting NK cells (*Granzyme K (GZMK), Cathepsin O (CTS0), -K (CTSK), -S (CTSS) *and *-Z (CTSZ)*), but they were rapidly downregulated upon stimulation with IL2. A rapid induction of other molecules involved in granule mediated cytotoxicity were detected concurrently (*Granzyme B (GZMB), Granulysin (GNLY), Perforin 1(PRF1), Cathepsin D (CTSD), Cathepsin C (CTSC/DPP1) *and *TIA1*). Late activation was observed for *Granzyme A (GZMA), -M (GZMM), -H (GZMH), Cathepsin W (CTSW), -B (CTSB) *and -F *(CTSF) *(Figure 2A and additional file 6(A)).

Many members of the TNFSF and TNFRSF were upregulated early. Some affecting the death receptor apoptotic pathway (*TNFSF6 *(*FASL*) and *TNFSF10 (TRAIL)*) and others probably activating the NF-κB pathway (*TNFRSF1B (TNFRB), TNFRSF14, TNFRSF4, TNFα (late activated*) and *TRADD (late activated)*). Of interest was *TNFSRF14*, which can interact with several members of TRAFs to activate innate immune response in concordance with increased expression of several members of the TRAF family (*1, 2, 3, 5 *and *7*). In addition, a group of of dipeptidylpeptidases (*DPP 1, 3, 7 *and *8*) (*DPP4 *and *10 *in additional file 6(A)), most of which are serine proteases involved in cleaving proline adjacent bonds, showed increased expression mainly at 8–24 hours. The well characterized DPPs implicated in cytolysis are *DPP1 *(*Cathepsin C/CTSC*), involved in processing of GZMA, -B and DPP4 (CD26), a serine protease involved in CD16 mediated cytolysis by NK cells [21].

#### Cytokines, chemokines and their receptors

A few chemokine ligands and receptors showed high expression in resting cells (*CXCR4 *and *CCL28*) (Figure 2B) likely related to the immediate immune response of NK cells. A group of chemokine receptors (*CCR7, CCR1, CXCR3, CMKOR1 (CXCR7*) and *CX3CR1*) showed an early increased expression after IL2 stimulation. Later a large number of chemokines and a few chemokine receptors (*CCL3, CCL4, CCL5, Chemokine- like factor superfamily members (CKLFSF6, -8, -3, -7 *and *-4), CCR5, CCR6 *and *CXCR6*) showed increased expression at 8–24 hours (Figure 2B). The increased expression of these genes is likely responsible for the proinflammatory response of NK cells, including the recruitment of NK cells as well as other immune cells to sites of inflammation. Many interferons and interferon induced genes were upregulated *(IFNγ, IFN induced protein 44 like (IFI44L), IFNγ inducible protein 16(IFI16), IFN regulatory factor 6 (IRF6), -2, -7, IFNA16, -4, IFNAR1, -2, IFN induced protein 35 (IFI35), -44, -30, IFN induced protein with tetratricopeptide repeats 1 (IFIT1), IFIT3 *and *IFIT5) *(Figure 2B). Some of the interleukins (*IL8, -18 *and *-24) *and interleukin receptors (*IL7R, -21R, -12RB1, -8RB *and -*11RA*) were highly expressed in resting cells and decreased upon IL2 stimulation. Others were upregulated on IL2 stimulation (*LTA (TNFB*), *IL2Rα, IL2Rβ, IL18R1, IL17RB, IL12RB2, IL17R, IL2RG, IL15, IL32/NK4, IL16, IL27RA, IL15RA *and *IL18RAP*) (Figure 2B). This profile of IL and ILR expression suggests that resting NK cells are ready for immediate immune response by secreting a number of cytokines. After activation, many ILRs were expressed, indicating that activated NK cells show enhanced sensitivity to autocrine and/or paracrine stimulation by other cells recruited to the site of inflammation.

#### Secretory signature

The transport of nascent polypepetides synthesized in the endoplasmic reticulum (ER) to the Golgi apparatus is an important step in the secretion of mature proteins through exocytosis. Proper folding and appropriate modifications are also crucial steps in protein secretion. The gene profiles showed upregulation of large groups of genes involved in secretory functions. Many genes involved in modifications or modulations of proteins affecting their stability and activity were upregulated early (2 hours) (*DEGS1, PPP2CB *and *MGAT2) *and later (8–24 hours)(*FKBP11*) (Figure 2C and additional file 6(B)). A large group of genes involved in vesicle trafficking in the ER (*SSR3, SEC61G, 61A1, -24C *and *-23B*), post processing in ER for optimal enzyme activity (*CDKN3 late*) and cargo import to Golgi apparatus (*SLC3A2, -33A1, -30A5, -1A4, COPE *and *VDP*) were upregulated (Figure 2C). In the late phase of activation (24 hours), genes required for protein transport to specific destinations (*SRP9, SSR1, -4, RABAC1 *and *SPCS3)*, proper protein folding (*EDEM, HSPA5 *and *TXNDC5)*, modification (*DAD1, RPN1 *and *DDOST*) and receptor mediated processing *(PPIB*) were overexpressed.

#### Cell surface/adhesion molecules

**Figure 3:**
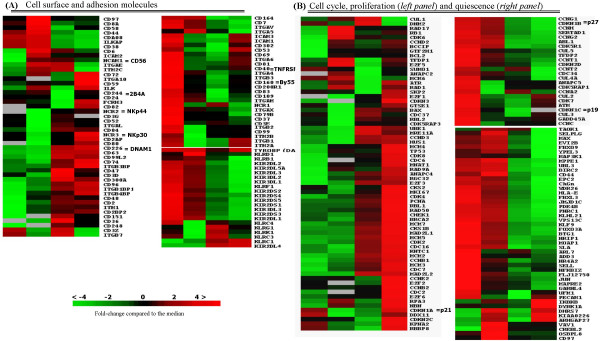
Differential gene expression between resting NK cells (column1) and NK cells after 2, 8 or 24 hours (colums 2, 3 & 4 respectively) of IL-2 activation are shown. A) Cell surface and adhesion molecules B) Cell cycle, proliferation and quiescence (*See also figure 2 legend for details*)

On IL2 stimulation, activating NK cell receptors or co-receptors (*CD226 (DNAM1), NKp44 (NCR2), NKp30 (NCR3), CD314 (KLRK1), KLRB1, KLRC3 *and *KLRC1*) showed increased expression and NK inhibitory receptors (*KIR2DL2, -DL3, DL1, KIR2DS4, -DS5, KIR3DL3, -DL1 *and -*DL2*) showed decreased expression (Figure 3A). However, it was not a universal shift towards an upregulation of activating receptors as some of the activating NK receptors (*NKp46 (NCR1), CD160 (By55), KLRC4 *and *KLRF1*) had dimished expression in activated cells. There was also increased expression of some inhibitory receptors (*CD300A (IRp60), KIR2DL4 *and *KLRG1*). A number of transcripts (*CD3Z, CD6, CD8A, CD97, CD58, CD56 (NCAM1), CD102 (ICAM2), CD44, CD38 and CD47*) were upregulated at 2–8 hours. The expression of these genes is generally related to lymphocyte activation or lymphocyte trafficking and cell adhesion. Many integrins (*ITGAV, ITGA6, -4, -B3, ITGAM, ITGAX, ITGB2 *and *ITGB1*) and other adhesion molecules (*CD302, ICAM1, CD208 (ICAM3) *and *CD81*) had high expression in resting cells which are likely important for NK cell homing. The late induced (24 hours) surface molecules included *CD96 (NK cell activating receptor TACTILE*), *CD63 (Granulophysin*), *integrin alpha L (ITGAL) *and *integrin-β7 (ITGB7)*, that are all important for adhesive interaction of NK cells during immune responses.

#### Cell cycle and proliferation

In freshly isolated NK cells, there was high expression of a group of genes that had cell cyle regulating functions (Figure 3B). Thus, there was high expression of inhibitory interaction partners of CDKs (*CDKN1B (p27) *that prevents the activation of cyclin E-CDK2 or cyclin D-CDK4 complexes, *CDKN2D (p19) *inhibitor of CDK4 and *CDK5RAP1*, inhibitor of CDK5). Other negative cell cycle regulators (*CCNG2, CCNG1 *associated with G2/M phase arrest in response to DNA damage and Cullins (*CUL5, -4A, -2 *and *-3*)) had increased expression suggesting a tight control of cell cycle progression in the resting circulating NK cells of peripheral blood. A few activators were also present (*CDK5R1 (p35) *activator of CDK5, *CDK7 *that together with *CCNH *(high in resting cells) and MAT1 functions as a CDK-activating kinase (CAK) and *CCNC *that interacts with CDK8) (Figure 3B). In keeping with the postulate, these inhibitory genes showed rapid downregulation upon IL2 activation. Concomitantly, there was an early (2 hours) increase in expression of genes involved in regulating general transcription, initiation/regulation of DNA replication, DNA repair and translation control (*Cullin1, CDK8, RAD17, GTF2H1 *and *TFDP1*)(Figure 3B and additional file 6(C)(*TFDP2*)). Numerous genes involved in the G1/S phase of the cell cycle (inhibitors: CDKN1A (*p21) *and *BCCIP; activators CDC2, -7 *and *-6*), members of cell cycle transcriptional regulators (*E2F family member 1, -2, -3, RB1 *and *RBL2*)(*RBL1 *additional file 6(C)), and associated proteins (*CDK8, -4, -2 *and *6*) and many *cyclins (D3, B1, E2, B2 *and *D2*) showed an increased expression at 8–24 hours. A family of genes crucial for DNA replication in the S-phase (*MCM2,-3,-4 *and *-6)(MCM5*, additional file 6(C)) showed maximum expression at 24 hours. Most of the M phase and anaphase regulators were not upregulated. Interestingly, a few genes involved in the late phases of of cell cycle: anaphase promoting control *(ANAPC4*) and kintechore associated gene (*KNTC1*) were highly expressed by 24 hours, suggesting that they may serve some different functions at earlier phases of the cell cycle. The proliferation marker PCNA showed high expression by 24 hours (Figure 3B and additional file 6(C)).

### Signal transduction

#### JAK/STAT pathway

**Figure 4:**
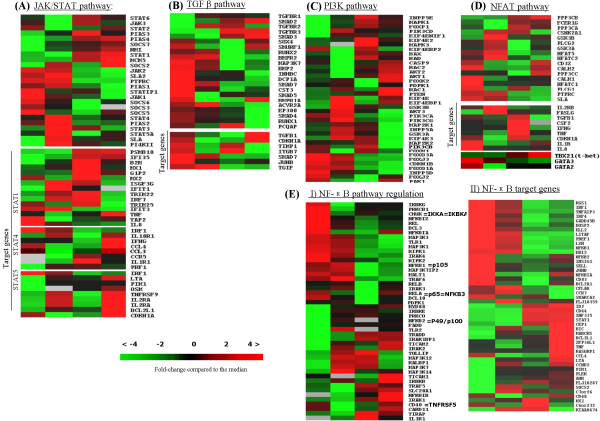
Signaling pathways. A) JAK/STAT pathway, B) TGFB1 pathway, C) PI3K-activation pathway, D) NFAT activation pathway, E) I: NF-κB pathway regulation and II: NF-κB target genes. (*See also figure 2 legend for details*)

Cytokine signals mediating control of cell growth and survival often involve the JAK/STAT pathway. Thus, addition of IL2 to resting NK cells in culture induced upregulation of an important upstream member of this pathway (*JAK3*) and major substrates (*STAT4 *and *-5A *[early phase], *STAT1 *and -6 [late phase]) and trancriptional mediators of STATs (*MCM5 *and *NMI*)(Figure 4A and additional file 7(A)). Several negative regulators of the JAK/STAT pathway: supressors of cytokine signaling (*SOCS3 *and *-6*) were downregulated after 2 hours with IL2 stimulation, whereas others (*PIAS2, -4 *and *SOCS7) *were upregulated. Activation of JAK2 and JAK3 in response to IL2 in NK cells has been reported previously and implicated in the activation of STAT4 and STAT5, respectively [22-24]. These observations are in agreement with our findings that 7 of 8 STAT4 target genes (when CCL3 is included) examined and 8 of 9 STAT5 targets (additional file 7(A)) showed increased expression in IL2 stimulated cell. Examination of the targets of STAT1 showed an increase in 13 of 15 target genes. The transcript levels of JAK1, STAT3 and STAT2 did not show a consistent pattern on the two platforms and they were not analyzed further.

**Figure 5:**
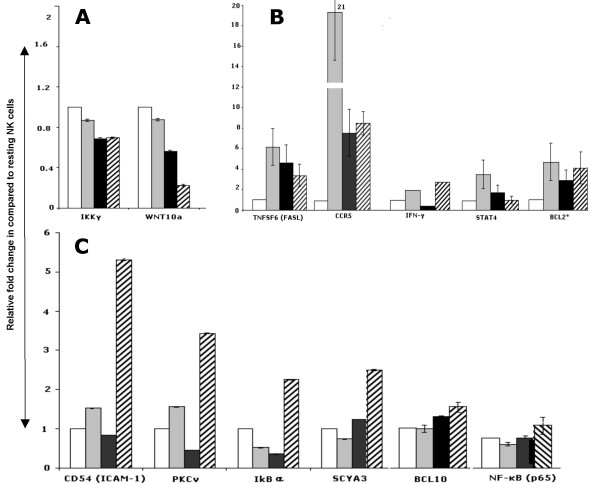
Confirmation of the microarray analysis with real time quantitative RT-PCR. The differential expression of some transcripts that have no previous reported association with NK biology were further validated by a real time quantitative RT-PCR. Temporal changes in the expression of selected genes from (A) Gene downregulated on IL2 activation (B) early upregulated genes (2 hours) and (C) late upregulated genes (8–24 hours).

#### TGFβ pathway

Transforming growth factor-β (TGFβ/TGFB1) pathway regulates diverse cellular function through SMAD proteins, which regulate transcription through their interaction with other transcription factors and the recruitment of co-repressors or co-activators depending on the cellular and functional context. Three members of TGFB1 type II receptors (*BMPRIA (ALK3), BMPR2 (t-ALK*) and *ACVR2A)*, several ligands (*BMP2*, *INHBC *and *TGFB1 *(TGFβ)) and many members of the SMAD family (R-Smad (*SMAD5*), Co-Smad (S*MAD4) *and I-Smad *(SMAD7*)) were upregulated in resting NK cells (Figure 4B). We also observed increased expression of SMAD4 interacting coactivators (EP300, DCP1A, and PCQAP) and marginal decrease in co-repressor *TGIF *expression in resting cells. SMAD ligases (*SMURF1*) and associated proteins (*RUNX1 *and *RUNX2*) showed increased expression in resting cells. The elaboration of TGFB1 protein by resting NK cells has been well documented and the downregulation of TGFβ pathway in NKcells activated by proinflamatory cytokines (24–48 stimulated hours) has also been observed by others [25]. We also observed a change in the expression profile of transcripts on this pathway on IL2 activation with downregulation of many of the transcripts mentioned above and upregulation of receptors (*TGFBR1*,*TGFBR2 *and *TGFBR3*) and the R-SMAD (*SMAD2*) in activated NK cells. *SMAD3 *showed brief marginal repression upon IL2 activation (2 hours), but upregulation in late activated cells (24 hours). Some negative regulators also seemed to be active in resting cells but examination of the target genes of TGFβ/SMADs showed high expression in 6 out of 7 genes. Thus, it appears that resting NK cells have an active TGFB1 signaling pathway involving at least SMAD4 and SMAD5 instead of SMAD2, features that are similar to signaling mediated by other members of the TGFβ superfamily, namely the BMPs [26]. An active TGFβ pathway may be important in maintaining growth arrest through inhibition of granulocyte-macrophage-colony-stimulating factors (GMCSF) [27] or induction of p21 and p15, in addition to the negative modulation of pro-inflammatory and cytolytic activites [25,28].

#### PI3K- pathway activation

Upon IL2 stimulation of NK cells, signal transduction from tyrosine phosphorylation of the IL2Rβ chain results in activation of the PI3K signaling pathway [29]. Three genes encoding PI3K catalytic subunits had highest expression levels after 2 hours (*PIK3CA, PIK3CD *and *PIK3CG*), while the transcript encoding the β-subunit showed a slower rise (*PIK3CB*). This increased PI3K expression occurred even though the negative regulator of the pathway, the phosphatase PTEN [30] showed progressively increased expression in the stimulated cell (Figure 4C and additional file 7(B)). PI3K activation enhances cell survival and antagonizes apoptosis via AKT of which subtypes (*AKT1 *and *-2) *became upregulated by IL2. Activated AKT inhibits apoptosis by phosphorylating BAD that is part of the BAD/BCLX_L _complex. Phosphorylated BAD dissociate from the BAD/BCLX_L _complex, thereby promoting cell survival. This correlates well with downregulated AKT target genes that are crucial for cell cycle regulation e.g. the *FOXO genes (3A *and *1A*) responsible for promoting quiescence [31] and *p27*, a cell cycle inhibitor. In addition, AKT activates EIF4E, a promoter of protein synthesis through mTOR and *EIF4E *had increased expression in activated cells. PI3K can also regulate cytotoxicity by activation of the RAC1, PAK1, MEK1/2 (MAP2K2) and ERK1/2 (MAPK1) pathways [9]. The expression of all these genes was upregulated in activated cells. Since *AKT1*and *-2*, *RAC1, PAK1, MEK1/2 (MAP2K2) *and *ERK1/2 (MAPK1) *were upregulated and *FOXO3A, -1A *and *p27 *were downregulated in our study, it is likely that PI3K influences several pathways simultaneously affecting cell survival, cell cycling and cytotoxicity.

#### NFAT activation in NK cells

The activation of NFAT is regulated by Ca^2+ ^/calcineurin dependent signaling and has been implicated in the secretion of various cytokines upon CD16 ligand binding in NK cells [32]. The transcriptional activity of NFAT can be activating or inhibitory, depending on the co-factors, including AP1(Fos/Jun), MEF2, GATA and histone deacetylases (HDACs). Among the five members of the NFAT family, only *NFAT5 *showed upregulation at the early phase of IL2 stimulation in both platforms (2 hours)(Figure 4D), similar to the report by Jin et.al (see figure 3B in [33]). In T cells, NFAT activation is mediated through the CD3ζ and CD3γ of the TCR and regulated by the phosphatase calcineurin. Calcineurin can dephosphorylate NFAT (1–4) proteins, leading to their nuclear import and DNA binding. The increased expression of *CD3ζ*, positive regulators of intracellular Ca^2+ ^release (*PLCγ 1 *and *-γ2*), catalytic calcineurin A subunits *(α (PPP3CA),β (PPP3CB) *and *γ (PPP3CC)) *and kinases for NFAT nuclear shuttling (*GSK3B, CK2(CSNK2A1) *and *JNK*) in activated NK cells suggested the induction of NFAT signaling on IL2 stimulation. Concordant with the above changes, we observed downregulation of crucial inhibitors (*SLA *and *PTPRC (CD45*)) of this pathway in activated cells. Several target genes were upregulated at different time points (Figure 4D), e.g. *FASL, IL2RB, CX3CR1 *(Figure 2B) and *TGFB1 *at 2 hours and *IFNγ, p21 (CDKN1A) *and *TNF2 *at 24 hours. A recent study showed that IL2 can induce CX3CR1 expression through NFAT2 (NFATC1) binding to its promoter, whereas IL15 represses it through induction of NFAT1 [34]. This observation indicates that NFAT1 (NFATC2) and NFAT2 (NFATC1) may have opposite roles in the expression of some genes in NK cells. NFATC1 (NFAT2) interacts with GATA3 [35], the major T-cell transcriptional regulator, that was highly expressed in resting NK cells as in naïve T cells and was downregulated on IL2 stimulation. *T-BET *(T-box expressed in T cells) on the other hand showed increased expression with IL2 stimulation. GATA3 is a Th2-regulating transcription factors that promotes the expression of IL4 and IL5, while T-BET is a Th1-specific transcription factor that controls the expression of CCR5, IFNG and IL18R1, all of which increased in activated NK-cells. These transcription factors may have similar roles in NK cells. T-BET is also required for terminal maturation and peripheral homeostasis of NK cells [36].

#### NF-κB pathway regulation

Resting cells have high expression of the NF-κB family genes (*REL, RELA (NFKB3 or p65), RELB *and *NF-κB1 (p105))*, some members of toll-like receptor (TLR) and IL1R pathway (Figure 4E, I and additional file 7(C)). Upon activation, maintained or increased expression of these transcripts was seen and many additional transcripts of different NF-κB activating signaling pathways were upregulated. Thus, in the TLR/IL1R pathway increased expression was observed for *TLR2 *and adaptor proteins (*Myd88, TIRAP, TICAM1 *and *TICAM2*), adaptor kinases (*IRAK1, -2 *and -*3*), kinase interacting protein (*Tollip*) and the kinases *TAK1(MAP3K7) *and *TAB2(MAP3K7IP2) *that activate the IKK complex. Members of the TNF receptor super family (*TNFRSF10A (death receptor 4) *and *TNFRSF6 (FAS)*), their adaptors protein (*TRADD*) and associated protein (*TRAF5*) also showed upregulation in activated cells suggesting TNF receptor mediated NF-κB activation since TRAF5 can recruit the IΦB kinases (IKKα, -β and -γ) to the TNF receptor complex, where RIP can activate the IKK catalytic subunits (IKKα and -β) through MAP3Kinases (Figure 4EI and additional file 7(C)). Furthermore, some of the positive regulators of the NF-κB signaling cascade in T cells through TCR signaling were also observed to be upregulated during both the early and late phase of IL2 stimulation. These included the kinases (*PKCθ *and *PDPK1) *involved in the activation of CARMA1 (*CARD11*) that leads to the phosphorylation of BCL10 (early down, then increasing) whereas the other essential component in T and B-cell antigen receptor mediated activation of NF-κB [37], MALT1 decreased. Since the expression of BCL10 varied between the two platforms the upregulation of BCL10 was confirmed by RT-PCR (Figure 5).

This upregulation of many genes in three different upstream pathways suggested multiple signals converged in the activation of the NF-κB pathway, perhaps exerting different influence at different time points. The downstream components of the pathway, which include NF-κB transcripts (*NF-κB1 (NFKB1 or p105), NF-κB2 (NFKB2 or p52/100) *and *RelB*) were also increased at 2 hours. NF-κB activity is negatively regulated by the binding of inhibitory proteins (IκB) of which *NF-κBIA *and *-Z (IκBα *and *-ζ*) showed decreased expression in activated cells, however *NF-κBIA *showed increased expression at 24 hours which correlated with the fact that NF-κB induces IκBα expression [38] (Figure 4E and 5). Phosphorylation of the IκB proteins by the serine-kinase complex (IκBKα (CHUK) IκBKβ, IκBKγ and IκBKε) marks them for destruction, thereby allowing the nuclear localization of the NF-κB dimers. Of these kinases *IκBKβ *and *IκBKε *showed marked increased expression whereas *IκBKα (CHUK) *and *IκBKγ *showed decreased expression in activated cells. Activation of NF-κB is supported by the expression of many target genes in IL2 stimulated cells at one or more time points (Figure 4E II and additional file 7(C)II). Interestingly, some of these genes showed high expression in freshly isolated cells. It is possible that the pathway was partially activated in resting cells, but it is also possible that some of the genes might be activated through the manipulation involved in the isolation of the NK cells. The expression of some NF-κB target genes e.g ICAM-1, IκBa, CCR5 and FASL was confirmed by RT-PCR (Figure 5).

## Discussion

A gene expression signature of resting NK cells from the peripheral blood of healthy donors and the changes in transcriptional profiles upon IL2 activation were obtained to overview the functional pathways underlying the biological properties of these cells. Others have addressed IL2 activation of NK cells for fixed time points of 4 hours [39] or 14 days with multiple activating stimuli with IL2, PHA and feeder cells [40] whereas our study is directed at early temporal regulation of pure NK cell activation. Our time course experiments demonstrated that IL2 exerts a broad range of effects on NK cells ranging from regulation of cell cycle, cell survival, cytotoxicity and secretion of immunologic and inflammatory effectors in a sequential manner. The use of two microarray platforms and independent NK cell populations validated the generated expression patterns and the biological properties that they suggested. Observed discrepancies between the two platforms indicate that technical variables and platform specific factors influence the large scale transcriptional profiles and sounds cautionary note for efforts to interpret differential gene expression. The technical variables include RNA amplifications protocols, which may also influence the gene expression profile [41]. We have used very strict criteria in our comparative analysis of the two platforms to attend greater accuracy but this approach may lead to the lost of some information. However, the basic information generated from these platforms correlates well when gene signatures and biological pathways rather than single genes were compared. It is also important to keep in mind that high expression of components of a signaling pathway does not indicate activation of that pathway which may involve phosphorylation, specific intracellular localization or other posttranslational determinants. On the other hand, when group of genes subserving specific functional activities show altered expression patterns, it is indicative perturbation of the pathway in response to a given stimulus.

*Resting NK cells *are characterized by a set of genes that maintain the cells at quiescent state as exemplified by the expression of *FOXO3A, SLA, KLF9 (BTEB1), PNRC1 *and *BTG1 *(Figure 3B* right panel*). An interesting finding is the high expression of many SMADs suggesting an active TGFβ pathway that may be part of the mechanism maintaining the resting profile and controlling the effector function of the NK cells. That these transcripts are involved in maintaining NK cells in the quiescent state is also supported by their rapid downregulation on IL-2 stimulation.

High expression of other effector-transcripts like cytotoxic effectors, cytokines and chemokines, NK receptors, unique surface markers and adhesion molecules illustrated the potential of circulating NK cells of the peripheral blood to catalyze and participate in the immediate immune responses. The presence of mRNAs encoding ligands like *CCL5, CXCL7, TNFSF14, FASL *and *CCL4 *might contribute to the killing of targets, activating other inflammatory cells and maintaining the circulating NK population in this reactive-prepared condition by autocrine stimulation loops. Thus, the CCR5 ligands *CCL5 *and *CCL4 *that are expressed in the resting NK cells may act directly on the growth and survival of neighboring NK cells expressing CCR5 at the initiation phase of an innate immune response. Such an autocrine loop is observed for CCL5-CCR5 in prostate cancer [42]. The effector expression profiles shift when the cells are stimulated and more receptor transcripts are expressed preparing the circulating NK cells to take on other functional roles and adapt to increased paracrine stimulation from other infiltrating immune cells.

Another interesting observation is the high expression of *GATA3 *in resting NK cells, similar to observation in resting T cells [43]. *T-BET*, on the other hand, had low expression in resting NK cells. There is evidence that both *GATA3 *and *T-BET *are important in the development of NK cells [36,44] but they may also be important in the function of mature NK cells. GATA3 is downregulated in Th1 cells, but its expression is maintained in Th2 cells. This raised the intriguing possibility that downregulation of *GATA3 *and upregulation of *T-BET *(Th1-specific transcription factor) in IL2 stimulated NK cells is required for the elaboration of Th1 type of cytokines in activated NK cells. Together with the decreased expression of *GATA3*, activated NK cells appear to change to a more Th1-like expression profile. While IL2 is a well known Th1 activator, a similar role has not previously been reported or observed for NK cells. The regulation of the transcriptional profiles of pro and anti inflammatory cytokines and chemokines through these transcription factors is an interesting area of future investigation.

*Stimulation of resting NK cells with IL2 *triggered an expression pattern consistent with the NK cells as important mediators of pro-inflammatory and innate immune response. Hence, the pro-inflammatory cytokines like *IFNγ, CCL5, CCL4, LTA *and *CCL3 *were upregulated whereas anti-inflammatory cytokines and receptors like *IL18BP *and *TNFRSF1B *[45,46] were downregulated. Combined with the activated innate immune response mediated by increased TLR signaling (*TLR2, Myd88, TIRAP, TICAM1, TICAM2*, *IRAK1, -2 *and -*3*, *Tollip*, *TRAF6, TAK1(MAP3K7) *and *TAB2(.MAP3K7IP2) *and the enhanced direct (*GZMA, PRF1, GZMB, GNLY *and *TNFSF6 (FASL*)) and indirect ERK enhanced (*PI3Ks, AKTs, RAC1 *and *MEK2 (MAP2K2*)) NK cell cytotoxicity [9] the stimulation of the circulating NK cells resulted in a significant shift in transcript profile reflecting the cells adapting to new functional roles. Strikingly, the cytolytic profile exhibited by activated NK cells resemble closely that of IL2 activated CD8+ T-cells [33]. In CD8+ T-cells, the cytotoxic effectors in granules (*GZMB, GNLY *and *PRF1*) and TNF family members (*FAS, LTA, TRAIL *and *TNF-α*) were induced whereas *GZMK *and *CD27 (TNRSF7*) were downregulated after 4 hours of stimulation (300 IU/ml IL2) (see figure 5B in [33]). In our study, the above mentioned genes were also upregulated but CD27 showed upregulation at 24 hours.

*IL2 stimulation mediated early activation of the JAK/STAT *signaling pathway (upregulated *JAK3, STAT3, STAT4, STAT5A*, and *STAT1*) hence affecting down stream transcription of many target genes (Figure 4A). When we examined STAT target gene expression, many targets of STAT1, -4 and -5 are upregulated providing confirmatory evidence of STAT1, -4 and -5 activation. Upregulation of JAK3, STAT-1, 3, and 5A was also observed by Jin and colleagues with purified CD8^+ ^and CD4^+ ^T cells stimulated with IL2 [33], indicating a common usuage of the JAK/STAT pathway in the activation in these cells, at least initially (see figure 1 in [33]). In our study the inhibitors of JAK/STAT signaling (*PIAS1, -2, SOCS3, -5 *and *-6*) were downregulated but *PIAS3, -4 *and *SOCS7 *were upregulated illustrating a balance that may limit the degree of JAK/STAT activation. IL2 can also activate the Ras-->RAF-->MEK-->ERK signaling pathway via JAK phosphorylation of SHC leading to stimulation of proliferation in T cells [47]. This may happened in NK cells and this is suggested by our study by the observed upregulated *MEK2 (MAP2K2) *and *ERK1 (MAPK1)*.

### Powerful pro-survival signals were induced by IL2

Four different genes of the PI3K family were upregulated as were the three isoforms of AKT kinases and simultaneously there was decreased expression of the AKT target genes (*FOXO1A, -3A *and *p27*). Together with the facts that activated AKT promotes cell survival through 1) phosphorylation dependent dissociation of the BAD/BCLX_L _complex [48] (Bad upregulated) and 2) activation of NF-κB through phosphorylation of IKK-β [49] (IKKβ upregulated) IL2 induced PI3K resulted in the expression patterns of transcripts that appear to promote survival and proliferation. Interestingly, when CTLs were simulated in the presence of other cells in PBMC, the upregulation of both PI3K and AKT were not detected[33] even though IL2 induced a general T cell activation and anti-apoptotic effect illustrating the importance of interactions between effector and bystander cells. Our study was focused on purified NK cells and the effects of bystander cells will not be observed.

NF-κB activation could be mediated by pathways other than IL2 induced PI3K activation, namely the TLR/IL1R pathway, the TNF pathway and possibly a NK specific surface receptor pathway involving BCL10. We have observed good evidence of NF-κB activation through the first two pathways. It is well established that in both T and B cells, BCL10 specifically mediate antigen receptor-induced NF-κB activation [37] In NK cells, BCL10 has been observed in the cytoplasm of normal NK cells, and in the nuclei of tumor cells of nasal NK/T-cell lymphomas [50]. Since we observed upregulation of *BCL10, NF-κB1 and NF-κB2 *upon stimulation of NK cells with IL2, it is possible that cytokine receptor mediated signaling also involve BCL10. The expression pattern of *CARD11*, a participant in BCL10 induced NF-κB activation in T and B cells [51] also supports this idea. In support of NF-κB activation was the upregulation of NF-κB1, NF-κB2 and the upregulation NF-κB target genes (Figure 4E II). In CD8+T cells, Jin et.al observed increased expression of several mediators of NF-κB pathway, possibly through modulation of TCR signaling (see figure 3 in [33])

### Activation of NFAT signaling pathway

PI3K can promote NFAT nuclear accumulation in two ways: by enhancing Ca^2+ ^mobilization and calcineurin-dependent NFAT dephosphorylation leading to nuclear import, or by AKT-mediated inactivation of GSK-3 that can phosphorylate NFAT and drive nuclear export [52]. The increased expression of positive regulators of intracellular Ca^2+ ^release, activators of calcineurin, catalytic calcineurin A subunits and kinases for NFAT nuclear shuttling in activated NK cells suggested both mechanisms to be involved in the NFAT signaling induced by IL2. *NFATC1 *showed highest expression in resting NK cells, similar to a prior observation in resting T cells, where it is the predominant NFAT protein [53]. NFATC1 interacts with GATA3 to maintain the differentiated Th2 phenotype [35]. This supports the notion of suppressed expression of Th1 type cytokines in resting NK cells. The profile shifted with the downregulation of *GATA3 *and upregulation of *T-BET *on activation.

### Temporal pattern of transcript expression

The early increase in transcription (within the first 2 hours) is likely regulated directly by signaling events – "first wave" induced transcription. Genes that were upregulated only after 8–24 hours are likely to represent activation of "second wave" genes and these were numerous in our study and especially represented genes involved in adhesion, secretory pathway, cytotoxicity and cell cycle control. These "second wave" transcripts are probably under the control of upstream genes or induced by factors such as cytokines or chemokines that are elaborated by the cells at a later time point as illustrated by general upregulation of target genes of STAT1 and NF-κB. Many genes had expression patterns where transcript levels were upregulated at 2 hours, low at 8 hours and then high again at 24 hours (genes important for cytotoxicity (*TIA1 *and *PAF1*), cytokine signaling (*IL2RA *and *IL18R1)*, secretory signaling *(SLC3A2, HM13 *and *DEGS1)*, cell cycle regulation (*MCM6 *and *CCND2))*. The control of transcription of these genes is more complex and may involve feedback inhibition or the induction of inhibitors. Time course experiments are therefore important to gain a more complete picture of the biology and function of the cell of interest.

The effect of IL2 on cytotoxic cells have been reported by several groups [16,33,39,54,55] showing many similarities as well as differences. For example, CD8^+ ^T cells stimulated with 300 IU/ml of IL2 for 4 hours either alone or cocultured with PBMC upregulate IL7, IL13, TNFα and IFNγ whereas the NK cells in our study did not express IL7 or IL13, and the upregulation of TNFα and IFNγ was registered at 8 hours. The cytokine profile of activated NK cells showed marked differences with that observed in activated CD8+T cells by Jin et.al[33], with the exception of a few genes (e.g. downregulation of IL8 and CXCR4, and upregulation of IL15 and IFN-γ). A few chemokines e.g.CCL3 and CCL4 were only observed to be upregulated at 24 hours in activated NK cells, whereas in CD8+ T cells, these chemokines are upregulated early (4 hours). On the other hand CCL5, IL16 and CXCR3 were downregulated in isolated CD8+ T cells, but upregulated in NK cells. In general, a detailed comparison is very difficult because of the different systems used and the different time point at which the assays were performed. The expression of many of the genes are highly variable over a 24 hr period so there is a significant degree of uncertainty when compared with results from a particular time point.

In most of the studies, the ethnic composition of the subjects studied was not specified but a systematic comparison of two ethnic groups was performed by Jin and co-workers [33]. The latter study is important in highlighting racial differences in immune response and may provide insight into the differences in disease susceptibility and response to immune modulation.

## Conclusion

*In conclusion*, our study demonstrated gene expression profiles associated with IL2 induced increased cytotoxicity, changes in chemokines, cytokines and adhesion properties, enhanced pro-inflammatory and innate immune response and changes in signaling pathways in NK cells. The changes in chemotactic signaling and surface/adhesion profile may enable the IL2 activated NK cells to migrate and infiltrate tissues where inflammation occur and upon arrival recruit other effector cells of the immune system. At the same time, NK cells may be more responsive and involved in both autocrine and paracrine signaling in the local environment than previously recognized. There is good evidence indicating NF-κB activation which may play a central role in pro-survival and pro-inflammatory function in activated NK cells. The role TGFβ in maintaining cell quiescence, GATA3/T-BET as master regulators of transcription of pro- and anti-inflammatory cytokines and BCL10 in NF-κB activation are intriguing and merit further investigation. NFAT signaling pathway also appears to have a functional role in IL2 stimulated NK cells. Future investigations including the interactions with other immune cells would provide a more complete picture of the possible changes of GEP on activation in various in vivo situations. Comparison of different ethnic groups especially with SNP data will allow better understanding of normal and disordered immune response.

## Methods

### Cell extraction and stimulation

Primary NK cells were isolated from seven healthy donors and each time point contained NK cells from 3 or 4 different donors (additional file 8). The healthy donors provided peripheral blood mononuclear cells (PBMCs) with IRB approval. PBMCs were isolated by Ficoll-Hypaque density gradient centrifugation at room temperature. Non-adherent cells were collected after incubation in nylon wool for 1 hour at 37°C and were mixed with anti CD3, CD14, CD19, CD36 and IgE MicroBeads (MiltenyiBiotec, Auburn, CA) at temperatures between 4–10°C for the negative selection of NK cells. NK cells with the CD56^+^/CD16^+ ^and CD3^- ^phenotype were negatively selected at temperatures between 4–10°C and re-examined by flow-cytometry to ensure purities greater than 90% (Figure 1). Purified NK cells were either treated immediately with Trizol (Invitrogen, Carlsbad, CA) or cultured in RPMI 1640 medium (Life Technologies, New York, NY) supplemented with 10% fetal bovine serum, 200 ug/ml streptomycin and 200 IU/ml penicillin, and IL2 (100 IU/ml) for 2, 8 or 24 hours at 37°C and 5% CO_2_, before harvest and storage in Trizol at -80°C till RNA isolation. Each healthy donor was represented in at least 3 different time points and each time point contained NK cells from 3 or 4 different donors. For an independent experiment, NK cells from six new healthy donors were purified and RNA from at least 3 different donors at each time point was pooled and hybridized on GeneChipU133 plus 2^® ^(Affymetrix Inc, CA). The purity of NK cells was determined by two-color flow cytometry with Alexa488-labeled monoclonal antibody (mAb) against CD3 and Alexa647-labeled mAb against CD56 or CD16. Of the negative selected cells 91% to 98% expressed CD56/CD16 but not CD3 (Figure 1). Cytospins of pre- and post purified PBMCs were stained with Wright-Giemsa stain for some samples to assess the enrichment of large granular lymphocytes.

### RNA extraction and T7 amplification

Total RNA was extracted with Trizol and further purified with RNeasy Mini Columns (Qiagen, Valencia, CA) before aliquots were run in agarose gel electrophoresis and measured by spectrophotometer at 260 and 280 nm to assess the quality of the RNA. For each time point, equal amounts of RNA from at least 3 different healthy donors were pooled prior to one round of RNA amplification using MessageAMP™ aRNA kit (Ambion, Austin, TX) according to the manufacturer's instruction. To minimize biases in RNA amplification only one round amplification was performed and using similar incubations times and 200 ng of total RNA of good quality as template for the reverse transcription reaction [56]. The quality and quantity of aRNA was monitored on agarose gel electrophoresis and by spectrophotometer. Typically, 10–20 ug of aRNA was generated from 200 ng of total RNA by one round of amplification and 10 ug of aRNA were employed for hybridization.

### Chemical labeling of aRNA

aRNA was chemically labeled with a platinum-linked cyanine dye using the MicroMax ASAP RNA labeling kit (PerkinElmer, Boston, MA) as per the manufactures instruction. Briefly, 10 ug of aRNA was incubated at 85°C for 15 minutes with labeling buffer and either Cy5 or Cy3 in a total volume of 20 ul prior to termination of the reaction by cooling on ice and addition of 5 ul of stop buffer. Labeled aRNA was purified on MicroCon 50 columns before the final volume was reduced to 3 ul by vacuum centrifuge (Savant, Farmingdale, NY). aRNA from NK cells was labeled with Cy5 whereas samples from similarly amplified lymphoid RNA reference standard, consisting of RNA from tonsil, thymus, spleen, and cell lines derived from malignant pre-B cells, plasma cells and NK cells [57] was labeled with Cy3. Two-color array analyses were used and determination of expression levels among samples was based on differences of ratios between sample and reference. Other reference RNA, including tonsillar, thymus and resting CD8^+^T was hybridized for comparative analysis, to obtain a signature for NK cells that is distinctive from cytotoxic T cells in addition to being distinctive from a general lymphocyte pool represented by the lymphoid standard.

### Hybridization, washing and scanning

Microarrays with 60-mer oligonucleotides (ONs) (Compugen, Jamesburg, NJ) printed on lysine-coated glass slides representing 17,260 genes, including reference (control) genes were utilized for hybridization. Labeled aRNA from NK cells and the lymphoid standard was combined in 6 ul of deionized water prior to the addition of poly (dA), human cot-1 DNA and yeast t-RNA at a final concentration of 10 ug each in a total volume of 45 ul containing 4.4 × SSC, 4.1 × Denharts and 50% formamide. Hybridization and washing were performed as described[57]. Arrays were scanned using an Axon 4000 scanner at 10 um resolution and images were analyzed using Genepix 5.0 software (Axon Instruments, Union City, CA). Raw gene expression data for spotted oligonucleotide arrays are available at [58](accession number .GSE8170)

### Gene expression profiling with Affymetrix chip (GeneChipU133plus 2^®^)

The isolation of RNA, hybridization and image processing was performed strictly according to the protocol set up by Affymetrix (Affymetrix Inc, CA). Briefly, extracted RNA (at least 2.6 μg used for each time point) was assessed by the Bio-analyzer 2100 (Agilent Inc, CA), and single round amplification protocol (one-cycle target labeling kit, Affymetrix Inc) was utilized for RNA amplification. Antisense biotin-labeled cRNA (10 μg), generated by *in vitro *transcription were hybridized and biotinylated probes hybridized to the array were detected using pycoerythrin labeled streptavidin. All arrays were scanned using the Gene-chip scanner 3000 (Affymetrix Inc, CA). The Affymetrix GeneChip^® ^Operating Software (GCOS) was utilized for management, sharing, and analysis of data generated. Other reference RNA was also profiled, namely lymphoid standard RNA [57], universal RNA standard and resting CD8^+ ^T cells. The data was analyzed using the same procedure, as above analysis and only those genes were selected which show similar trend of expression with the spotted array data. Raw gene expression data for affymetrix arrays are available at [58](accession number GSE8059)

### Data analysis

The fluorescent intensity levels corresponding to each hybridized DNA spot was analyzed with Genepix 5.0 software (Axon Instruments, Union City, CA), and hybridized spots having signal-to-noise ratios below 1.25 and spots with blemishes were flagged and removed prior to further analysis. Local background was subtracted from the individual spots and intensities for each channel were normalized with respect to median intensity from each channel for the entire array. The raw expression data from all the time series were uploaded in BRB-ArrayTools software (Biometric Research Branch-NCI, MA) and global normalization was used to median center the log-ratios on each array in order to adjust for differences in labeling intensities of the Cy3 and Cy5 dyes on different arrays. We excluded genes from the analysis showing minimal variation across time series experiments, and genes whose expression differed by at least 1.25 fold from the median in at least 20% of the arrays were retained. Adjusted data were further filtered to remove genes with opposite ratio values and genes differing more than 3 fold in duplicate analysis of the same NK sample. For SOM, (self organizing maps), the weighted average was calculated for each gene from the duplicate hybridizations according to the formula μ = Σ (*x*_i_/σ_i_^2^)/Σ(1/σ_i_^2^), where each data point *x*_i _is weighted inversely by its own variance σ_i_^2 ^[59]. The weighted average ratio between Cy5 and Cy3 were log_2 _transformed and arrays from the time series were centered by resetting the equality parameter (ratio = 1) to the mean of all the experiments before SOM analysis[60]. SOM, an artificial intelligence algorithm based on unsupervised learning configures output vectors into a topological presentation of the original data. Parameters with similar features are mapped to the same map unit or nearby units in a SOM that allows various visual inspections of the clustering of microarray data (additional file 5).

The pathway analysis was performed using the pathway analytical tool of the BRB-ArrayTools (NCI, NIH-MA) software [61]. In this tool, two statistics are computed that summarize the p-values for groups of genes in a pathway; the Fisher (LS) statistic and the Kolmogorov-Smirnov (KS) statistic as described [18] We considered a pathway significant differentially regulated if either significance level was less than 0.001 or at least 5 genes of a pathway are represented on the array.

For the supervised pathway analysis, the genes for selected pathway were downloaded from the Superarray database (SuperarrayInc, MD) and the from the immune signature database [19]. The normalized expression of the selected genes was extracted and differentially expressed genes were as those that had at least a 2-fold alteration in expression level between the time points analyzed. Other selected genes were grouped according to their functional characteristics available through OMIM database)[62], Entrez Gene [63] and Pubmed [64]databases. Similar procedure was used for data analysis on GeneChipU133plus2^® ^and only those genes were selected for further analysis which show similar trend of expression with the spotted array data.

### Real time quantitative PCR

To confirm the differential mRNA expression identified by microarray assay, an independent NK cell isolation, flow cytometry validation of purity and IL2 stimulation was performed. A total of 13 genes were selected for validation by SYBR Green real time quantitative RT-PCR. In brief, 200 ng RNA was reversely transcribed into cDNA with 200 ng random hexamer using MMLV-RNase H^- ^reverse transcriptase as per the manufacturer's instructions (Invitrogen, Carlsbad, CA). The human actin, RPL13a and UBC transcripts were used to normalize the expression levels of genes across different time points for comparative analysis. The primers were designed to amplify the cDNA close to the 3' end of the transcript and all the PCR products were less than 200 bp in length. Quantifications were done in triplicate and mean values and standard deviation were calculated for each transcript.

## Authors' contributions

KD carried out the NK isolation procedure, IL-2 stimulation, in vitro RNA amplification, and microarray experiments and drafted the manuscript. JI participated in the design of the study, microarray procedure, data analysis with HG and LX and drafted the manuscript. GZ carried out the confirmatory analysis of microarray experiments through quantitative real time-PCR and various technical assistance and advice. AS ensured high purity of isolated NK cells by flow cytometry evaluation. FD participated in helpful discussions, interpretation of the data and provided financial support to KD. WCC conceived, organized and supervised the study, and participated in the analysis and interpretation of the data.

## Supplementary Material

Additional file 1 Correlation Coefficient Mapping. Reproducibility of the duplicate hybridization experiments on spotted microarray was checked through correlation coefficient mapping programmed in BRB-ArrayTools. High correlation is seen among technical duplicates from the same samples. Click here for file

Additional file 2 NK signature dervived from oligonucleotide microarray platform. Differentially expressed genes with ≥ 2 fold higher expressions in resting NK cells compared to the lymphoid RNA standard and also ≥ 2 fold higher expression than tonsillar cells and resting CD8^+ ^T cells. Click here for file

Additional file 3 NK signature dervived from affymetrix chip platform. The NK cell signature derived from the GeneChipU133plus 2^® ^was based on similar comparisons as performed in spotted microarray, with the modification that, tonsil profile was replaced with a universal RNA standard (Stratagene Inc, CA). Click here for file

Additional file 4 Common genes in resting NK signature from signature database [17] and our Affymetrix data. NK signature common to our data set and curated signature from the signature database (Shaffer, A.L., et al. Immunol Rev, 210, 67–85, 2006) Click here for file

Additional file 5 SOM representation of the genome wide transcriptional changes upon IL2 stimulation. The NK0 map represent resting NK cells, NK2, NK8 and NK24 maps represent NK cells after 2, 8, or 24 hours of culture with IL2, respectively. Color coding index stands for the expression values of genes so that the brighter the color, the higher the value. Each hexagon in a certain position of the maps contains a group of genes, identified by the SOM algorithm that has very similar expression patterns throughout the time points of the experiment. Differentially expressed genes are illustrated: (A) Early upregulated, (B) Early downregulated, (C) Genes upregulated at 8 hours, (D)Genes downregulated at 8 hours, (E) Late upregulated, (F) Late downregulated, (G) Genes showing continuous increased expression during culture period. Click here for file

Additional file 6 Representative groups of genes or pathways with sufficient expressed common genes for both are presented showing similarty in both formats. The maximum differences in expression levels between resting NK cells and IL2 activated NK cells after 2, 8 or 24 hours for the spotted microarray data are shown. The color change in each row represents the gene expression relative to the median across the samples and values are visualized according to the scale bar that represents the expression fold (log2) relative to median. A) Cytolytic pathway, B) Secretory signature, C) Cell cycle and proliferation, D) Quiescent signature. Click here for file

Additional file 7 Signaling pathways. A) JAK/STAT pathway, B) PI3K-activation pathway, C) I: NF-κB pathway regulation and II: NF-κB target genes. (See also additional file 6 legend for details). Click here for file

Additional file 8 Supplemental Table 2: Donors of NK cells used in the experiments. Purity of the NK cells is indicated in () after the donor ID. Information on donor number and purity of included NK cells. Click here for file
