# Supplementary figures and images for: Genome wide transcriptional analysis of resting and IL2 activated human natural killer cells: gene expression signatures indicative of novel molecular signaling pathways
Source: BMC Genomics. 2007 Jul 10;8:230. doi: 10.1186/1471-2164-8-230 (PMC1959522; doi:10.1186/1471-2164-8-230)

## Slide 1
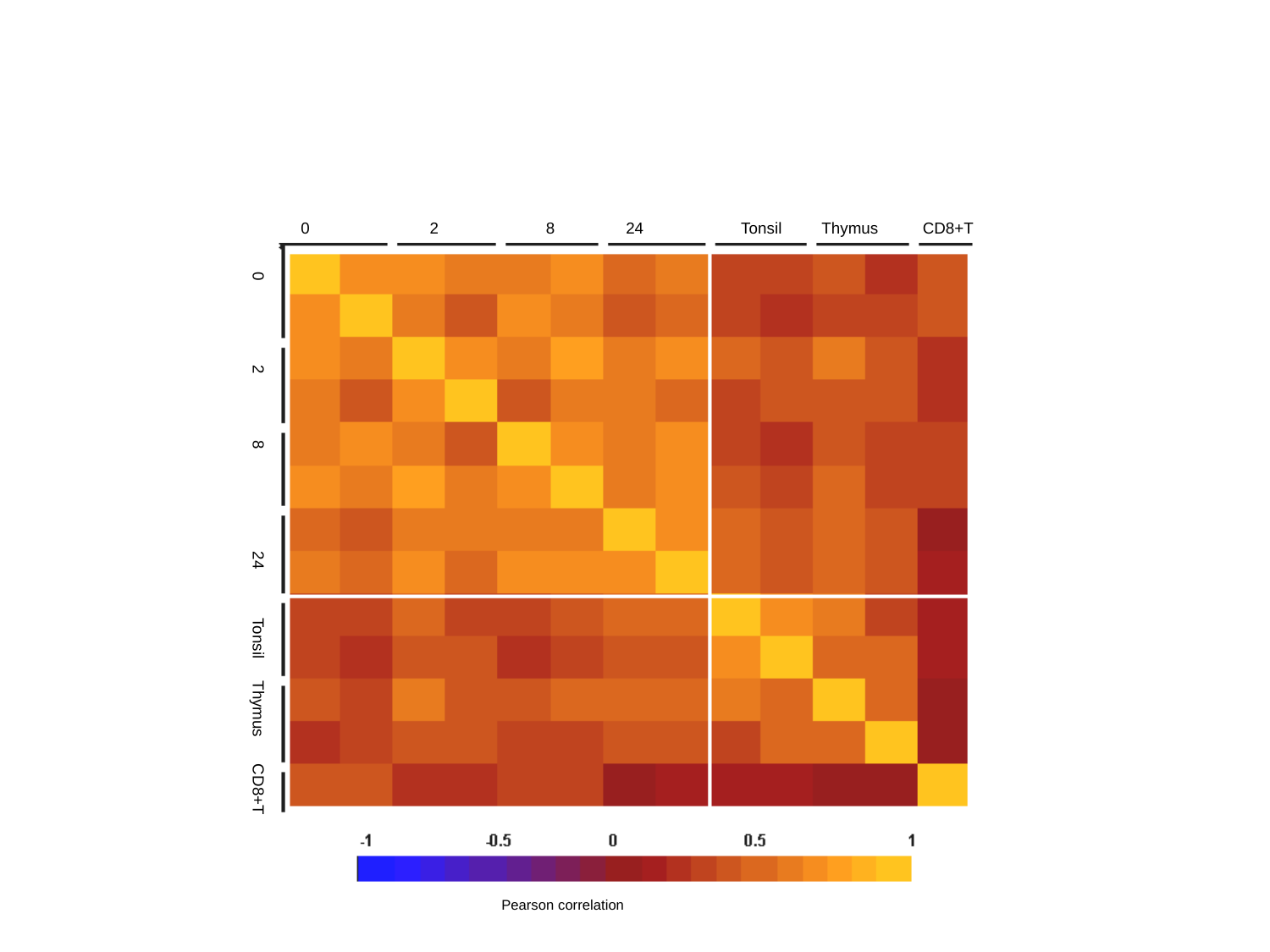

0 2	 8 24 Tonsil Thymus CD8+T
 0 2 8 24 Tonsil Thymus CD8+T
Pearson correlation

Supplement: Additional file 1 — Correlation Coefficient Mapping. Reproducibility of the duplicate hybridization experiments on spotted microarray was checked through correlation coefficient mapping programmed in BRB-ArrayTools. High correlation is seen among technical duplicates from the same samples. [file 1471-2164-8-230-S1.ppt]

## Slide 1
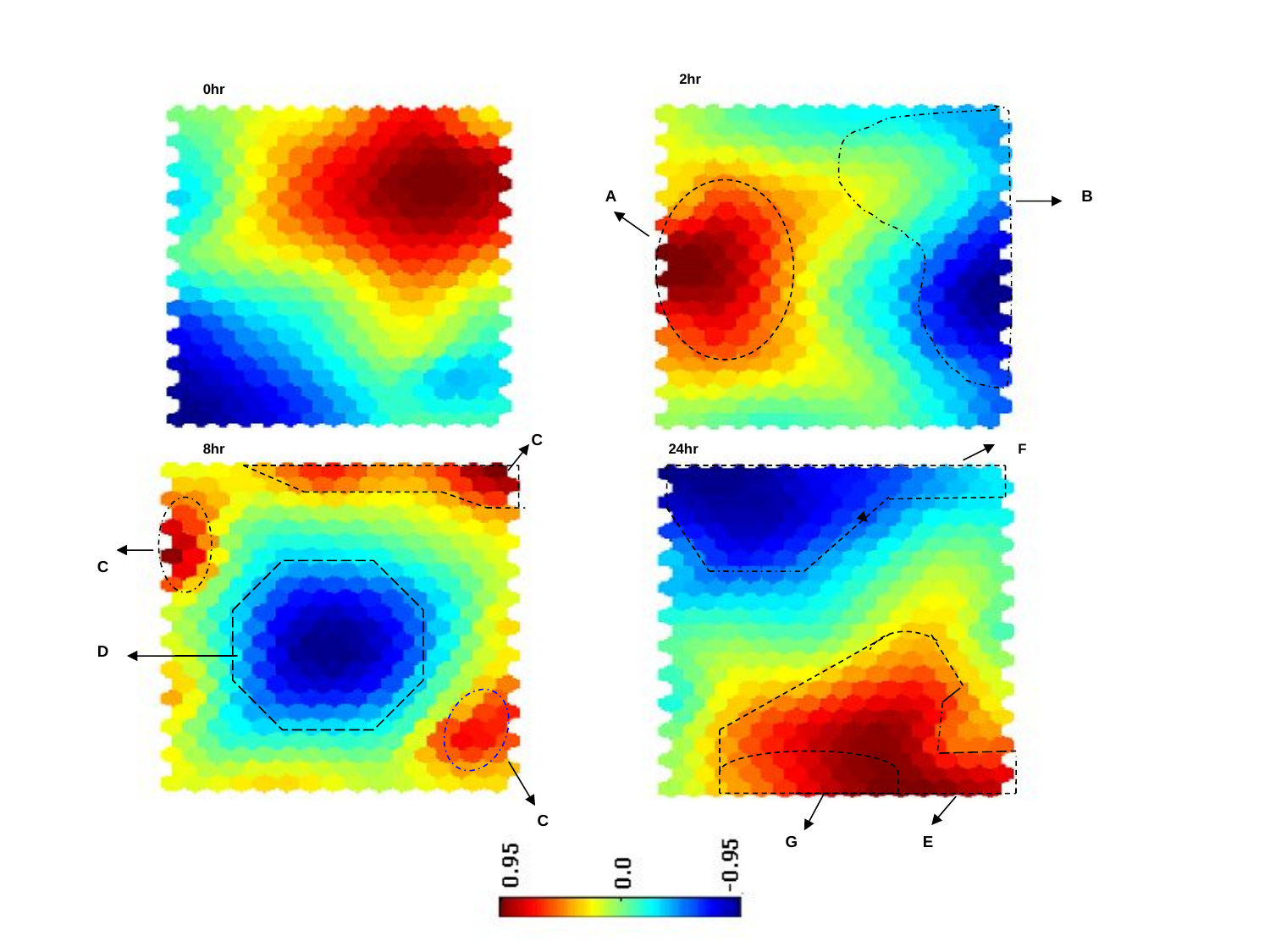

2hr
0hr
A
B
C
8hr
24hr
F
C
D
C
G
E

Supplement: Additional file 5 — SOM representation of the genome wide transcriptional changes upon IL2 stimulation. The NK0 map represent resting NK cells, NK2, NK8 and NK24 maps represent NK cells after 2, 8, or 24 hours of culture with IL2, respectively. Color coding index stands for the expression values of genes so that the brighter the color, the higher the value. Each hexagon in a certain position of the maps contains a group of genes, identified by the SOM algorithm that has very similar expression patterns throughout the time points of the experiment. Differentially expressed genes are illustrated: (A) Early upregulated, (B) Early downregulated, (C) Genes upregulated at 8 hours, (D)Genes downregulated at 8 hours, (E) Late upregulated, (F) Late downregulated, (G) Genes showing continuous increased expression during culture period. [file 1471-2164-8-230-S5.ppt]
