# Supplementary material for: Genome wide transcriptional analysis of resting and IL2 activated human natural killer cells: gene expression signatures indicative of novel molecular signaling pathways
Source: BMC Genomics. 2007 Jul 10;8:230. doi: 10.1186/1471-2164-8-230 (PMC1959522; doi:10.1186/1471-2164-8-230)
Supplement: Additional file 4 — Common genes in resting NK signature from signature database [17] and our Affymetrix data. NK signature common to our data set and curated signature from the signature database (Shaffer, A.L., et al. Immunol Rev, 210, 67–85, 2006) [file 1471-2164-8-230-S4.doc]

**Supplemental table 1**: NK signature common to our data set and curated signature from the signature database (Shaffer, A.L., et,al *Immunol Rev,* **210,** 67-85, 2006)

| **Unigene ID** | **Name** | **Symbol** | **Cytoband** |
| --- | --- | --- | --- |
| Hs.75703 | Chemokine (C-C motif) ligand 4 | CCL4 | 17q12 |
| Hs.81743 | CD160 antigen | CD160 | 1q21.1 |
| Hs.143212 | Cystatin F (leukocystatin) | CST7 | 20p11.21 |
| Hs.79226 | Fasciculation and elongation protein zeta 1 (zygin I) | FEZ1 | 11q24.2 |
| Hs.15125 | Hypothetical protein FLJ20699 | FLJ20699 | 22q13 |
| Hs.105806 | Granulysin | GNLY | 2p12-q11 |
| Hs.6527 | G protein-coupled receptor 56 | GPR56 | 16q12.2-q21 |
| Hs.1051 | Granzyme H (cathepsin G-like 2, protein h-CCPX) | GZMH | 14q11.2 |
| Hs.158315 | Interleukin 18 receptor accessory protein | IL18RAP | 2p24.3-p24.1 |
| Hs.75596 | Interleukin 2 receptor, beta | IL2RB | 22q13|22q13.1 |
| Hs.274601 | Killer cell immunoglobulin-like receptor, three domains, long cytoplasmic tail, 1 | KIR3DL1 | 19q13.4 |
| Hs.41682 | Killer cell lectin-like receptor subfamily D, member 1 | KLRD1 | 12p13 |
| Hs.183125 | Killer cell lectin-like receptor subfamily F, member 1 | KLRF1 | 12p12.3-13.2 |
| Hs.43803 | Leukocyte-associated Ig-like receptor 2 | LAIR2 | 19q13.4 |
| Hs.51305 | V-maf musculoaponeurotic fibrosarcoma oncogene homolog F (avian) | MAFF | 22q13.1 |
| Hs.79227 | Myomesin (M-protein) 2, 165kDa | MYOM2 | 8p23.3 |
| Hs.10306 | Natural killer cell group 7 sequence | NKG7 | 19q13.41 |
| Hs.44036 | Platelet derived growth factor D | PDGFD | 11q22.3 |
| Hs.198037 | Pleckstrin homology domain containing, family G (with RhoGef domain) member 3 | PLEKHG3 | 14q23.3 |
| Hs.2200 | Perforin 1 (pore forming protein) | PRF1 | 10q22 |
| Hs.325820 | Protease, serine, 23 | PRSS23 | 11q14.1 |
| Hs.158326 | Prostaglandin D2 receptor (DP) | PTGDR | 14q22.1 |
| Hs.199248 | Prostaglandin E receptor 4 (subtype EP4) | PTGER4 | 5p13.1 |
| Hs.62 | Protein tyrosine phosphatase, non-receptor type 12 | PTPN12 | 7q11.23 |
| Hs.144840 | Regulator of G-protein signalling 3 | RGS3 | 9q32 |
| Hs.170019 | Runt-related transcription factor 3 | RUNX3 | 1p36 |
| Hs.100602 | SMAD, mothers against DPP homolog 7 (Drosophila) | SMAD7 | 18q21.1 |
| Hs.288126 | Spondin 2, extracellular matrix protein | SPON2 | 4p16.3 |
| Hs.8182 | Spectrin repeat containing, nuclear envelope 1 | SYNE1 | 6q25 |
| Hs.272409 | T-box 21 | TBX21 | 17q21.32 |
| Hs.102866 | Transketolase-like 1 | TKTL1 | Xq28 |
| Hs.71791 | Yippee-like 1 (Drosophila) | YPEL1 | 22q11.2 |
