# Supplementary material for: Genome wide transcriptional analysis of resting and IL2 activated human natural killer cells: gene expression signatures indicative of novel molecular signaling pathways
Source: BMC Genomics. 2007 Jul 10;8:230. doi: 10.1186/1471-2164-8-230 (PMC1959522; doi:10.1186/1471-2164-8-230)
Supplement: Additional file 6 — Representative groups of genes or pathways with sufficient expressed common genes for both are presented showing similarty in both formats. The maximum differences in expression levels between resting NK cells and IL2 activated NK cells after 2, 8 or 24 hours for the spotted microarray data are shown. The color change in each row represents the gene expression relative to the median across the samples and values are visualized according to the scale bar that represents the expression fold (log2) relative to median. A) Cytolytic pathway, B) Secretory signature, C) Cell cycle and proliferation, D) Quiescent signature. [file 1471-2164-8-230-S6.ppt]

## Slide 1
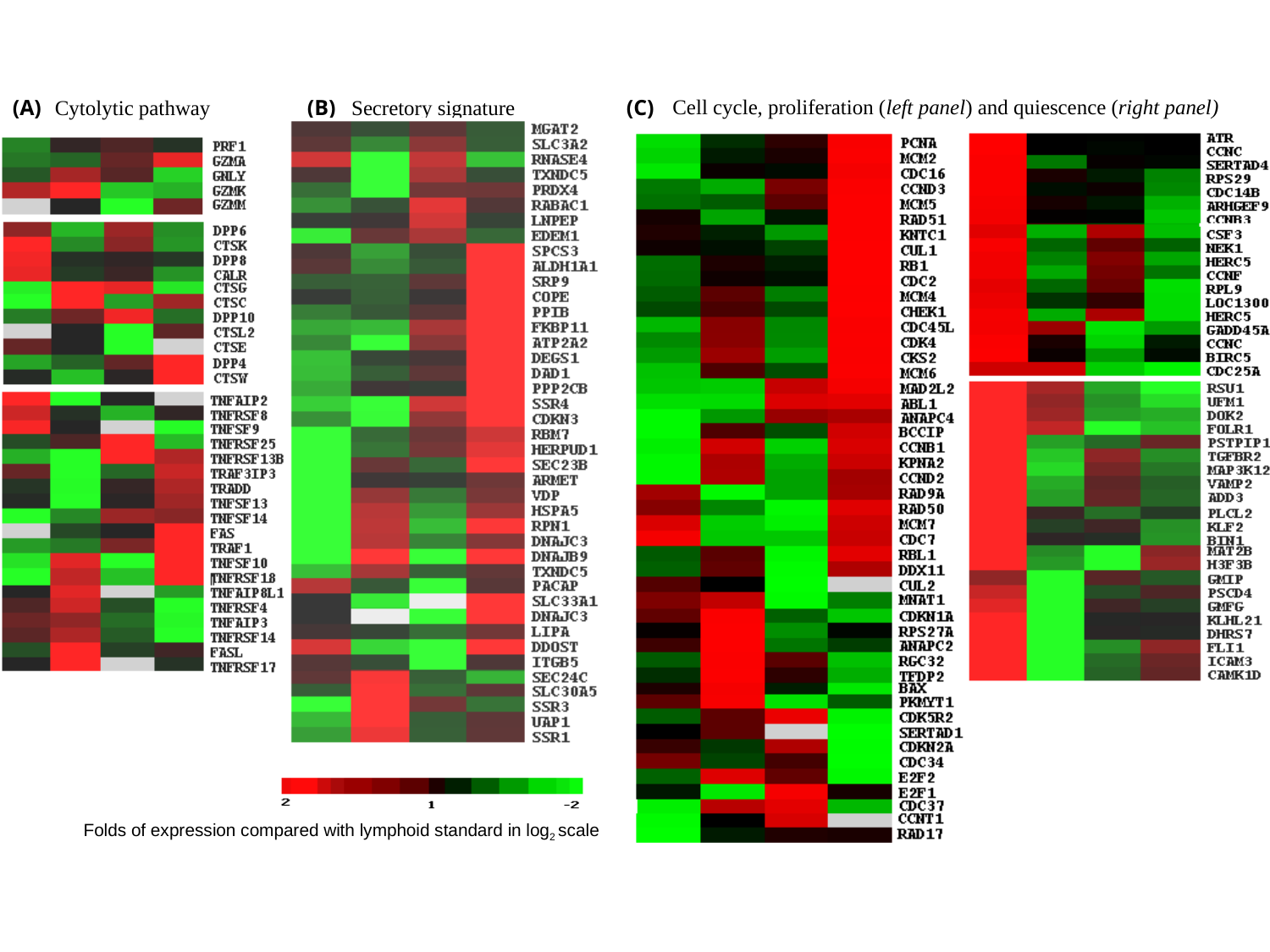

Cell cycle, proliferation (left panel) and quiescence (right panel)
(A)
Cytolytic pathway
(B)
Secretory signature
(C)
Folds of expression compared with lymphoid standard in log2 scale
