# Supplementary material for: Genome wide transcriptional analysis of resting and IL2 activated human natural killer cells: gene expression signatures indicative of novel molecular signaling pathways
Source: BMC Genomics. 2007 Jul 10;8:230. doi: 10.1186/1471-2164-8-230 (PMC1959522; doi:10.1186/1471-2164-8-230)
Supplement: Additional file 7 — Signaling pathways. A) JAK/STAT pathway, B) PI3K-activation pathway, C) I: NF-κB pathway regulation and II: NF-κB target genes. (See also additional file 6 legend for details). [file 1471-2164-8-230-S7.ppt]

## Slide 1
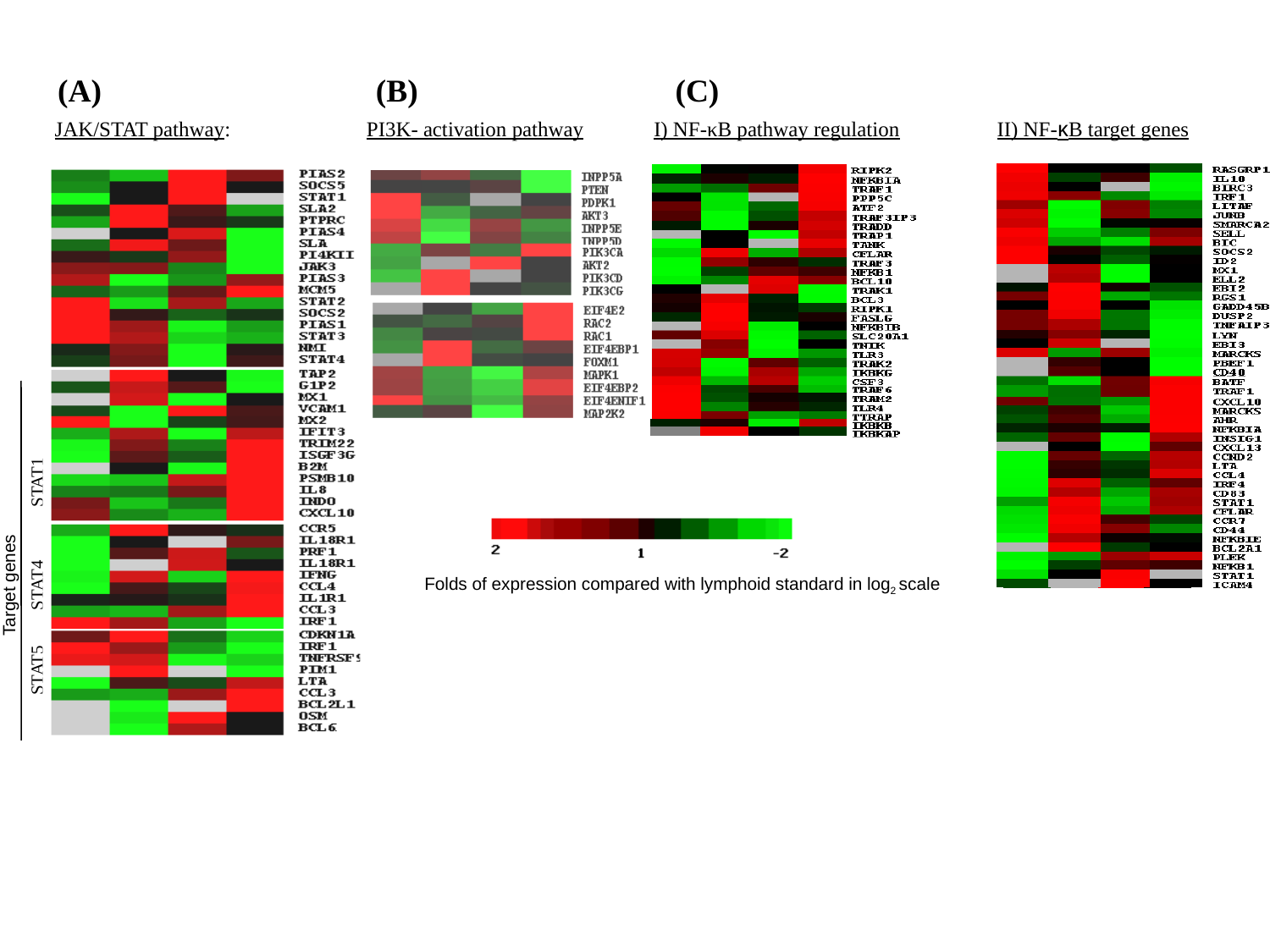

(A)		 (B) (C)
JAK/STAT pathway:
I) NF-κB pathway regulation
II) NF-κB target genes
PI3K- activation pathway
Target genes
STAT5 STAT4 STAT1
Folds of expression compared with lymphoid standard in log2 scale
