# Supplementary material for: Genome wide transcriptional analysis of resting and IL2 activated human natural killer cells: gene expression signatures indicative of novel molecular signaling pathways
Source: BMC Genomics. 2007 Jul 10;8:230. doi: 10.1186/1471-2164-8-230 (PMC1959522; doi:10.1186/1471-2164-8-230)
Supplement: Additional file 8 — Supplemental Table 2: Donors of NK cells used in the experiments. Purity of the NK cells is indicated in () after the donor ID. Information on donor number and purity of included NK cells. [file 1471-2164-8-230-S8.doc]

**Supplemental table 2: Healthy donor NK cells used in the experiments.**

| *(A****)Oligonucleotide Microarray*** | | | | | | | |
| --- | --- | --- | --- | --- | --- | --- | --- |
| *0 hours* | 2011(97.4) | 207(94.5) | 2606 (96.3) |  |  |  |  |
| *2 hours* |  | 207(94.5) | 2606 (96.3) | 1812 (91.1) | 512 (92.9) |  |  |
| *8 hours* |  |  | 2606 (96.3) |  | 512 (92.9) | 2311 (95.7) |  |
| *24 hours* |  |  | 2606 (96.3) |  | 512 (92.9) | 2311 (95.7) | 1906 (93.4) |
| ***(B)Affymetrix Microarray*** | | | | | | | |
| *0 hours* | 2012 (93.9) | 401 (93.0) | 202 (95.7) | 2202 (96.4) |  |  |  |
| *2 hours* | 2012 (93.9) | 401(93.0) | 202 (95.7) |  |  | 707 (93.7) |  |
| *8 hours* |  | 401(93.0) |  | 2202 (96.4) | 2906 (91.6) | 707 (93.7) |  |
| *24 hours* |  | 401(93.0) | 202 (95.7) |  | 2906 (91.6) | 707 (93.7) |  |
